# The Effects of the COVID-19 Pandemic on Health and Well-Being of Children and Youth in Nova Scotia: Youth and Parent Perspectives

**DOI:** 10.3389/fped.2021.725439

**Published:** 2021-11-17

**Authors:** Hilary A. T. Caldwell, Camille L. Hancock Friesen, Sara F. L. Kirk

**Affiliations:** ^1^Healthy Populations Institute, Dalhousie University, Halifax, NS, Canada; ^2^Division of Pediatric Cardiothoracic Surgery, Children's Health and Medical Centre, Omaha, NE, United States; ^3^School of Health and Human Performance, Faculty of Health, Dalhousie University, Halifax, NS, Canada; ^4^MacEeachen Institute for Public Policy and Governance, Dalhousie University, Halifax, NS, Canada

**Keywords:** student, mental health, feelings, physical activity, screen time, sleep, school, lockdown

## Abstract

**Objectives:** The COVID-19 pandemic led to school closures, cancellations of major events, and loss of in-person social interactions for children and youth. These restrictions undoubtedly impacted the lives of children and youth. This study describes the well-being of children and youth in Nova Scotia during the COVID-19 pandemic and their thoughts and feelings about the return to school, from the perspectives of both youth and parents.

**Methods:** A province-wide survey was conducted in August 2020 with parents of school-age children and youth and youth to measure youth well-being since the pandemic began.

**Results:** Parents of children and youth in grades pre-primary to 12 (*n* = 699; 53% girls) and youth in grades 3–12 (*n* = 279; 69% girls) completed the online survey. Perceptions of parents about children's emotions during the pandemic were: bored, safe, lonely, happy, and anxious. Youth reported feeling bored, relaxed, depressed, safe, and worried. Sixty-three percent of youth and 72% of parents reported that they/their child felt they were missing important life events. Parents reported that being with parents, being physically active and being with friends made their child feel positive. Youth reported that being with friends, pets and watching TV made them feel good during this time. Seventy-six percent of parents and 62% of youth reported they/their child were getting more screen time than before the pandemic. With schools closed, participants most frequently shared that they missed friends and social interactions, in-person learning, and extra-curricular activities. Youth and parents expressed worries about COVID-19 outbreaks and related restrictions when schools re-opened to in-person learning.

**Conclusion:** The well-being of children and youth in Nova Scotia was greatly impacted by the COVID-19 pandemic and related school closures in 2020. It is essential that pandemic recovery plans prioritize the health and well-being of children and youth.

## Introduction

The first COVID-19 cases were detected in Nova Scotia, Canada in March 2020. Schools were closed for in-person learning from mid-March until the start of the 2020–2021 school year in September. Children and youth were not able to participate in in-person extra-curricular activities, like music or sports during a province-wide state-of-emergency. Many outdoor spaces, like parks and trails, were also closed to discourage gathering (1). These measures were deemed necessary to stop the spread of COVID-19, but undoubtedly had impacts on the health and well-being of children and youth.

Well-being is challenging to define as individuals have different concepts and experiences of well-being (2). For example, it can be defined on a continuum from negative to positive and operationalized at either the individual or environmental level (3). Individual well-being can be categorized into five domains: physical, psychological, cognitive, social, and economic. The physical, cognitive, economic and social domains tend to measure positive indicators of well-being while the psychological domain mainly measures negative states, such as anxiety or depression (3). UNICEF conceptualizes child and youth well-being across nine domains: we are happy and respected, we are protected, we are participating, we are free to play, we are healthy, we are learning, we belong, we are secure and we are connected to the environment (2). Despite the many definitions of well-being, evidence suggests that high-quality peer relationships and supportive schools are associated with higher well-being, while exposure to psychosocial stress is associated with poorer well-being in children and youth (4).

When surveyed independently, parents and their children offer unique perspective on a child's health or well-being. For example, the Pediatric Quality of Life Inventory is a valid and reliable assessment of children's health-related quality of life that can be completed as a child self-report survey or by a parent-proxy (5). Parent-proxy reports have been justified because it was believed that children did not have the cognitive skills to self-assess; however, if tools are designed appropriately, children as young as 5 years old are able to self-assess their own quality of life (6, 7). Despite children's ability to self-report their own quality of life measures, it is still valuable to capture both child parent perspectives as these perspectives can affect decisions about access to services or healthcare for the child (7). It has also been reported that parent-child agreement on the assessment of child mental health problems is low (8). The link or agreement between parent and child perspectives of health and well-being may be associated with parenting styles. It has been reported that authoritative parenting, including respecting the child's autonomy and exercising parental authority over the child when necessary, is beneficial to children's mental health (9). Given the reported differences in parent-proxy and self-reports of children's quality of life and mental health and possible impact of parenting style on mental health, it is important to capture both parent and child perspectives for a more holistic assessment of a child.

Early studies have reported the burden of COVID-19 and related restrictions on child well-being in Canada and the United States. In a survey conducted by UNICEF Canada, youth reported missing being able to leave the house, go to school and spend time with friends (10). A study from the United States conducted daily surveys from Spring 2020 of service workers with young children. Many families experienced hardships during the crisis, including job loss, income loss, caregiving burden and illness. Early in the COVID-19 pandemic, reports of parental and child well-being were inversely associated with the number of crisis-related hardships experienced; a higher number of hardships was associated with lower well-being (11). Another US survey revealed 14% of parents reported worsening behavioral health. One in 10 parents reported that both their mental health and their children's behavioral health worsened during the pandemic (12).

It has been suggested that school closures related to COVID-19 may have negatively impacted child and youth well-being. In addition to education, schools provide essential health services and supports to students. For example, students may access school food programs, counseling services and vaccinations at school. It is also anticipated that students will need more non-academic supports when they return to in-person learning at school (13). Only 39% of grade 7–12 students in Nova Scotia reported that online learning was positive some or all of the time and 28% reported that they could not keep a routine for online learning (14). Given the exceptional situation of an extended school-closure, the Nova Scotia school re-opening plan recognized the need to focus on student well-being, including daily physical activity, time outside and building strong inter-personal relationships (15). For schools to re-open, new policies, such as mask wearing and physical distancing, were implemented (15). These anticipated changes at schools provided an opportunity to capture the thoughts and feelings of children and youth about their return to school.

The purpose of this study was to describe the feelings of children and youth, or their parents, about their health and well-being during the COVID-19 pandemic, and their feelings about return to in-person school in Nova Scotia. The child and youth parental perspectives were examined independently, and differences between parents and children and youth responses are described.

## Materials and Methods

### Participants

The study recruited children and youth in grades Primary to Grade 12 (age 5–18 years; hereinafter referred to as youth). In the case of youth in elementary school (grades pre-primary-−6, approx. age 5–11 years), who may have been too young to complete a survey, parents were invited to complete it on the child's behalf. For youth in junior high and high school (grades 7–12, approximate age 11–18 years), the youth were invited to complete the survey themselves.

### Survey Administration

The two versions of the survey (youth and parent) were administered online using Dalhousie University's Opinio software platform in August 2020. The Dalhousie University Research Ethics Board provided ethical approval for this study (#2020-5238). Participants were recruited through social media (Facebook, Twitter, Instagram) and through organizations that work directly with youth in Nova Scotia.

### Survey

The survey included both close-ended and open-ended questions. The close-ended questions included demographics, health behaviors (physical activity, sleep, screen time) and thoughts and feelings about the COVID-19 pandemic, being at home and the return to school. The open-ended questions asked about what students missed at school, supports from school and their hopes about the return to in-person learning in Fall 2020.

### Statistical Analyses

Quantitative data analyses were conducted with SPSS Version 26 (Armonk, NY: IBM Corp). Counts and proportions were used to describe responses to close-ended questions. Chi-square tests were used to determine differences in proportions between groups (i.e., parents vs. children and youth, rural vs. urban). Qualitative data analyses were conducted with NVivo Version 12.6.1 Plus (QSR International Pty Ltd.). Qualitative thematic analysis was used to identify themes with open-ended question responses. Qualitative responses were coded to identify and define emerging themes from the participants' responses. Themes were subsequently summarized using counts. Parent and youth survey responses were analyzed separately; however, differences and similarities between groups are presented.

## Results

The survey was completed separately by parents (*n* = 699; 53% female) and youth (*n* = 279; 69% female). Descriptive characteristics are included in Table 1. Parents' children were in grades pre-primary-12 and youth were in grades 3–12. Most participants lived in urban areas (66–78%), identified as European ethnicity (64–67%) and 66–77% lived with two parents in the same household.

**Table 1:** Parent and youth demographics.

	**Parents *n = 699***	**Youth *n = 279***
Grades, n (%)		
Pre-Primary-2	166 (23.8%)	
3–6	270 (38.6%)	11 (3.9%)
7–9	166 (23.8%)	77 (40.3%)
10–12	97 (13.9%)	191 (68.5%)
Community, n (%)		
Rural	232 (33.6%)	57 (21.8%)
Urban	458 (66.4%)	204 (78.2%)
Lived Gender, n (%)		
Girl	368 (52.5%)	191 (68.5%)
Boy	322 (45.9%)	73 (26.2%)
Trans Boy	2 (0.3%)	4 (1.4%)
Trans Girl	0 (0%)	1 (0.4%)
Gender non-binary	3 (0.4%)	3 (1.1%)
Two-spirited	0	2 (0.7%)
Other	2 (0.3%)	1 (0.4%)
Prefer not to answer	4 (0.6%)	4 (1.4%)
Household income		
< $49,999	156 (22.3%)	34 (12.5%)
$50,000–99,999	214 (30.6%)	69 (25.4%)
$100,000+	243 (34.7%)	45 (16.5%)
Prefer not to answer/ don't know	87 (12.4%)	124 (45.6%)

### Thoughts and Feelings About the COVID-19 Pandemic and Being at Home

**Figure 1:**
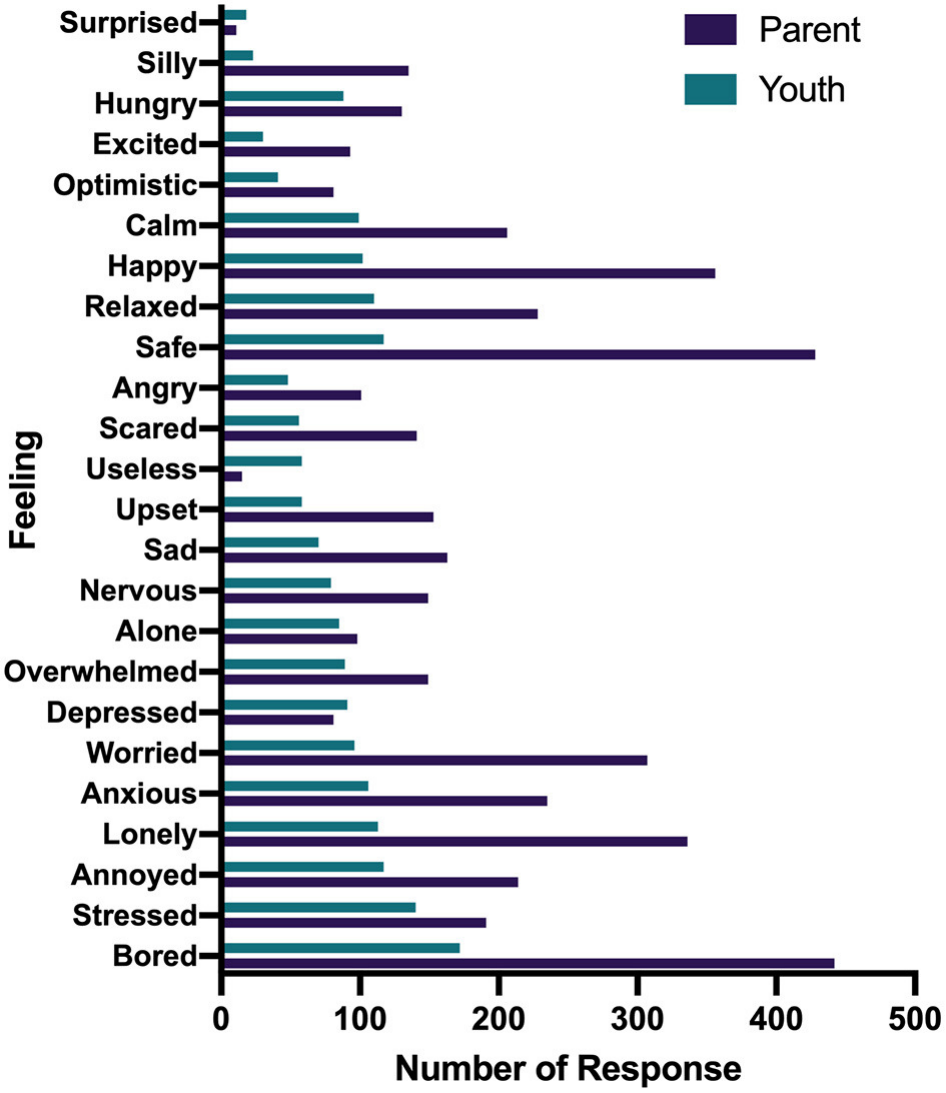
Reports of how child has been feeling since being home from school reported by parents and youth.

**Figure 2:**
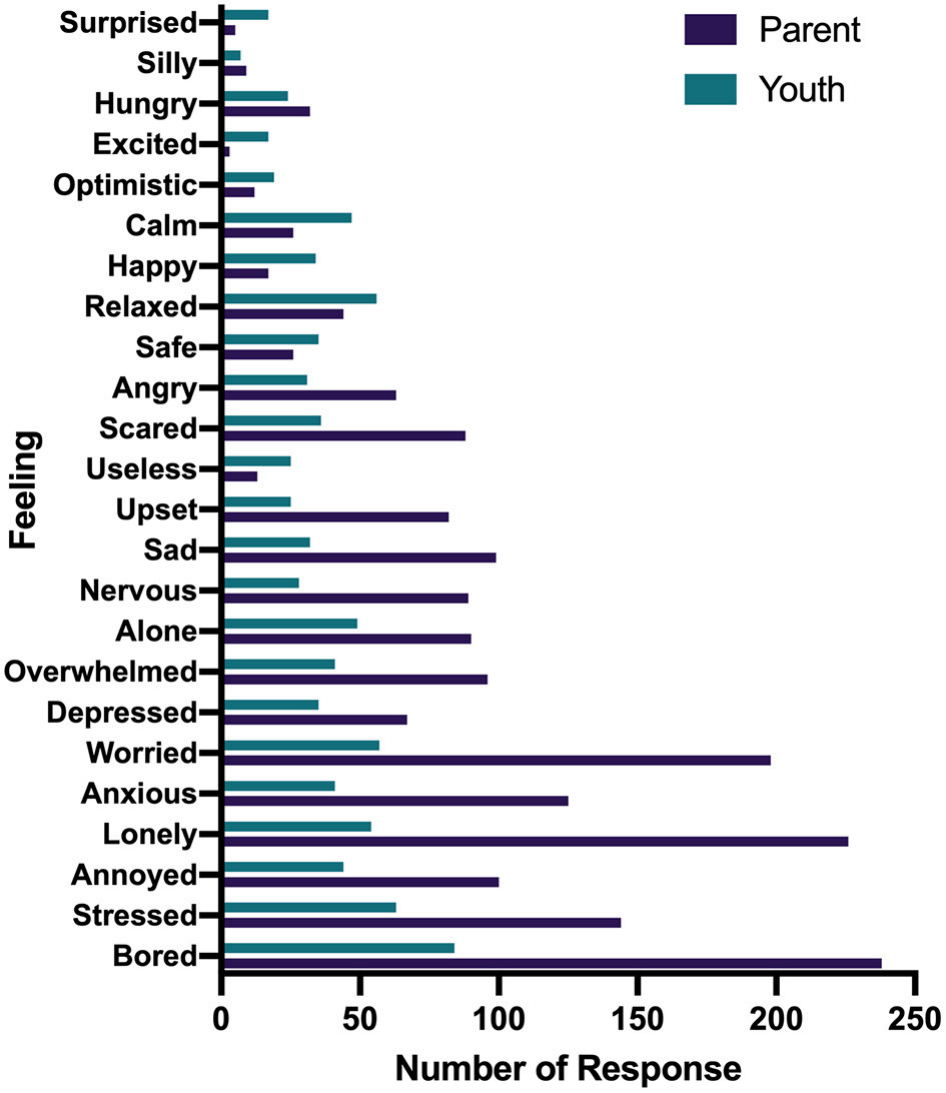
Reports how new feelings since being home from school reported by parents and youth.

**Figure 3:**
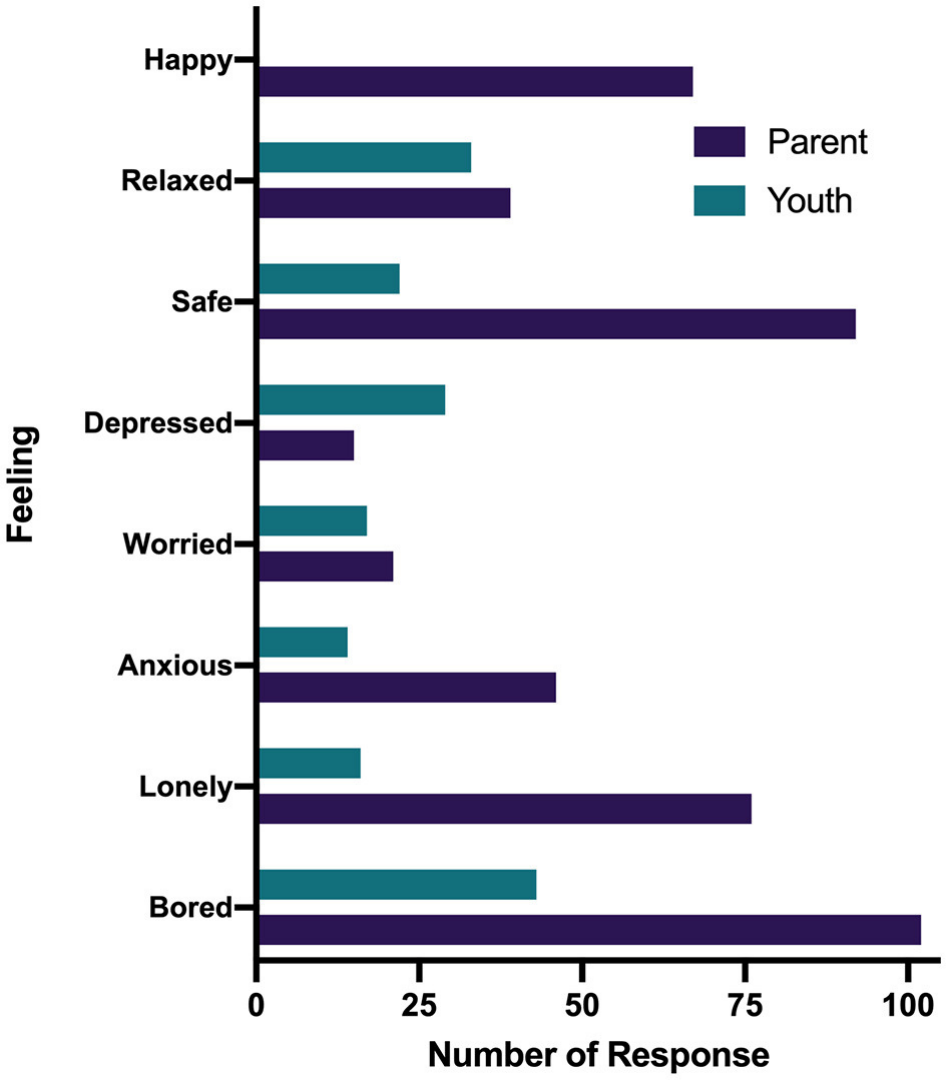
Most prominent feeling during the COVID-19 pandemic reported by parents and youth.

Parents and youth responded to the questions “how has your child/how have you been feeling since being home from school because of the COVID-19 pandemic?” and “what feelings would you say are new since schools shut down?” by selecting all feelings that were relevant (Figures 1, 2). The most expressed feelings since being away from school for children (reported by parents) were bored, safe, happy, lonely, and anxious. Youth most reported feeling bored, stressed, annoyed, safe, and relaxed. The most frequently reported new feelings about children by parents were bored, lonely, worried, stressed, and anxious while youth reported bored, stressed, worried, lonely, and alone. For the question, “which feeling was the most prominent during the lockdown?,” parents and youth selected one response only. The most prominent feelings reported by parents about their children were bored, safe, lonely, happy, and anxious and youth reported bored, relaxed, depressed, safe, and worried (Figure 3).

Parents and youth reported their level of agreement with statements about thoughts and feelings related to COVID-19 (Table 2). The distribution of responses between parents and youth was different for each question (*p* < 0.05), though absolute differences in proportions were minimal for some questions. Most parents (72%) and youth (73%) agreed or strongly agreed that they felt like they were missing important life events or moments because of COVID-19. Most parents agreed or strongly agreed that their child had a friend they can talk to about how they are feeling (67%) or had a pet who helps them feel better (75%), while 63% of youth agreed or strongly agreed they had a friend to talk to and 83% had a pet that helps them feel better. Most parents (68%) and youth (74%) agreed or strongly agreed that their child or they thought about how others are feeling during COVID-19.

**Table 2:** Thoughts and feelings about COVID-19 reported by parents and youth.

	**Strongly agree**	**Agree**	**Neither agree nor disagree**	**Disagree**	**Strongly disagree**	**Chi-Square Statistic, *p*-value**
Feels like they are missing important life events or moments because of COVID-19						
Parent	209 (29.8%)	293 (41.8%)	115 (16.4%)	66 (9.4%)	18 (2.6%)	*χ^2^* = 44.6, *p* < 0.001
Youth	120 (44.1%)	78 (28.7%)	35 (12.8%)	25 (9.2%)	14 (5.1%)	
Has a friend they can talk to about how they are feeling						
Parent	134 (19.1%)	338 (48.2%)	129 (18.4%)	87 (12.4%)	13 (1.9%)	*χ^2^* = 56.6, p <0.001
Youth	94 (34.3%)	105 (38.3%)	30 (10.9%)	28 (10.2%)	17 (6.2%)	
Has a pet who helps them feel better (if applicable)						
Parent	272 (39.7%)	243 (35.5%)	86 (12.6%)	29 (4.2%)	55 (8.0%)	*χ^2^* = 50.1, *p* < 0.001
Youth	118 (47.8%)	88 (35.6%)	25 (10.1%)	10 (4.0%)	6 (2.4%)	
Thinks about how others are feeling during COVID-19						
Parent	159 (22.7%)	315 (44.9%)	159 (22.7%)	51 (7.3%)	17 (2.4%)	*χ^2^* = 21.4, *p* = 0.001
Youth	76 (27.7%)	128 (46.7%)	45 (16.4%)	22 (8.0%)	3 (1.1%)	

### What Things Make Children and Youth Feel Good at This Time?

Parents and youth reported what things make children and youth feel good at the time the survey was completed (August 2020). The most prominent responses from parents about children were being with parents (*n* = 524, 75.0%), being physically active (*n* = 411, 58.7%), being with friends (*n* = 394, 56.4%), playing or being creative (*n* = 367, 52.5%) and pets (*n* = 357, 51.1%). The most prominent youth responses were being with friends (*n* = 167, 59.9%), pets (*n* = 145, 52.0%), watching TV (*n* = 134, 48.0%), cooking (*n* = 118, 42.3%) and being with parents (*n* = 117, 41.9%).

### Healthy Movement Behaviors

**Figure 4:**
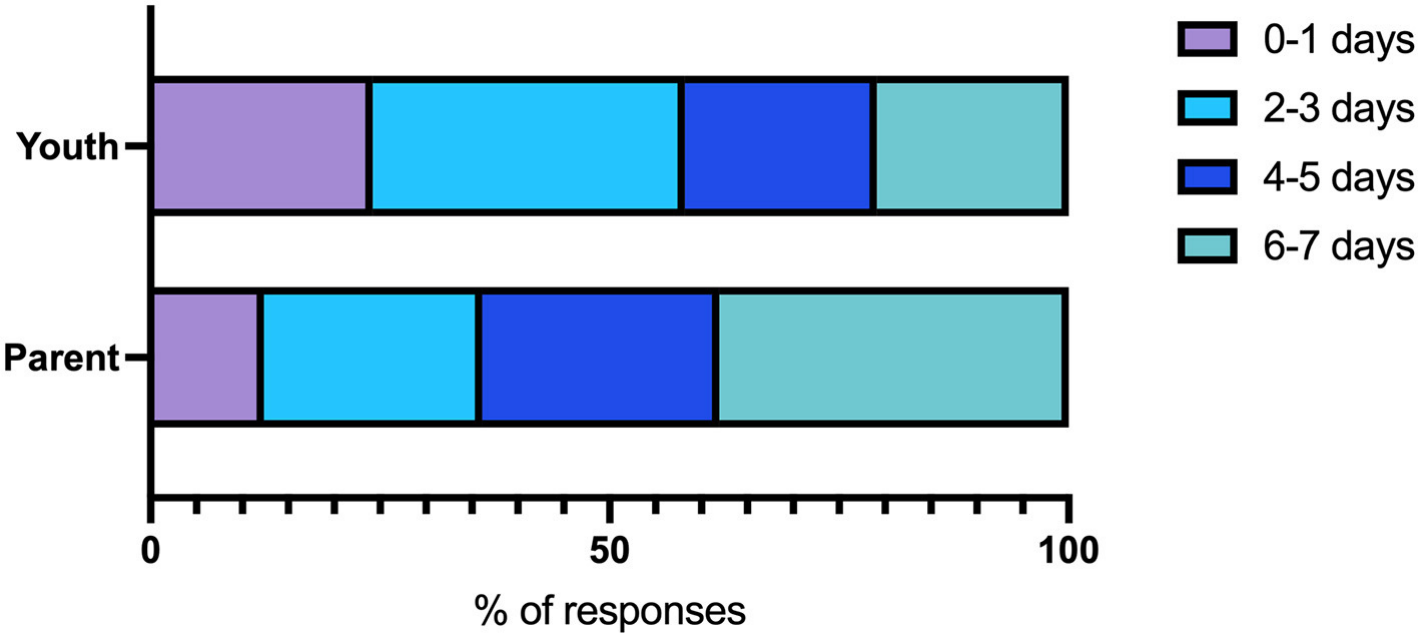
Number of days children and youth played, did sports, or exercised for at least one h in the last week as reported by children and youth or parents.

Changes (less, about the same or more) in health behaviors reported by parents and youth are provided in Table 3. Distributions of responses were different between children and youth (*p* < 0.05) but patterns were similar. Parents reported a higher proportion of children and youth being active for at least an hour on at least four days per week (*n* = 440, 63.8%) compared to youth-reported data (*n* = 112, 44.1%) (Figure 4). Parents reported their children were doing less (44%), about the same (40%) or more (17%) physical activity compared to before the pandemic, while 45% of youth reported less, 30% reported the same and 25% reported more physical activity. Most parents and youth reported that the same amount of space for play and other activities (that they could easily walk or cycle to from home) was available as compared to before the pandemic (Table 2). Parents and youth reported how safe their child, or they, felt playing in their neighborhood, with 1 indicating extremely unsafe and 10 meaning extremely safe. Seventy percent of parents and 53.9% of youth reported at least 8 out of 10, indicating they felt very safe in their neighborhoods during the pandemic.

**Table 3:** Changes in healthy behaviors compared to pre-pandemic.

	**Parents [n(%)]**	**Youth [n(%)]**	**Chi-Square Statistic**
Physical Activity			*χ^2^* = 18.9, *p* < 0.001
Less	305 (43.8%)	123 (45.1%)	
About the same	276 (39.6%)	82 (30.0%)	
More	116 (16.6%)	68 (24.9%)	
Spaces for play and other activities that you can walk or cycle to from home			*χ^2^* = 14.4, *p* = 0.006
Less	189 (27.1%)	79 (28.7%)	
About the same	485 (69.6%)	179 (65.1%)	
More	23 (3.3%)	17 (6.2%)	
Sleep			*χ^2^* = 71.0, *p* < 0.001
Less	78 (11.2%)	45 (16.4%)	
About the same	427 (61.1%)	90 (32.7%)	
More	194 (27.8%)	140 (50.9%)	
Screen Time			*χ^2^* = 40.2 *p* < 0.001
Less	16 (3.0%)	23 (8.4%)	
About the same	150 (28.0%)	81 (29.5%)	
More	535 (76.3%)	171 (62.2%)	

**Figure 5:**
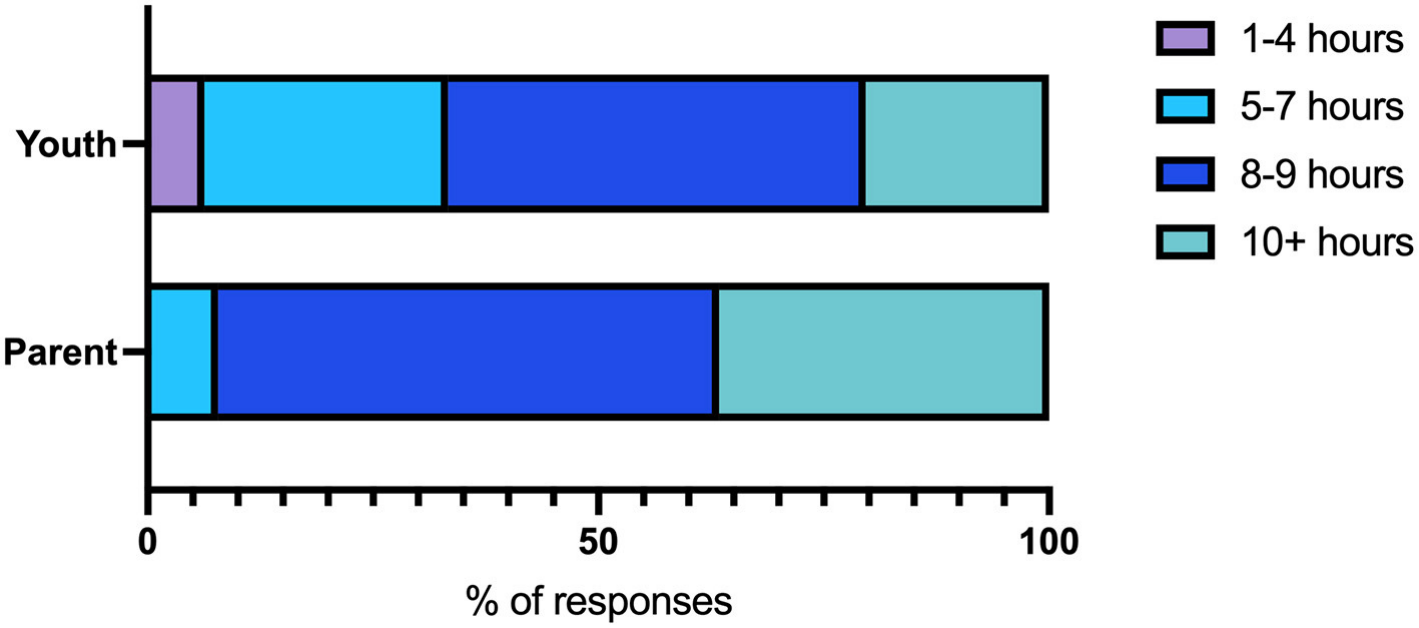
Number of hours of sleep within a 24-h period as reported by children and youth or parents.

The largest proportions of children and youth reported sleeping 8 or more h within a 24-h period (Figure 5). Sixty-one percent of parents reported this as about the same and half of youth reported this as more sleep than before the pandemic started (Table 3).

**Figure 6:**
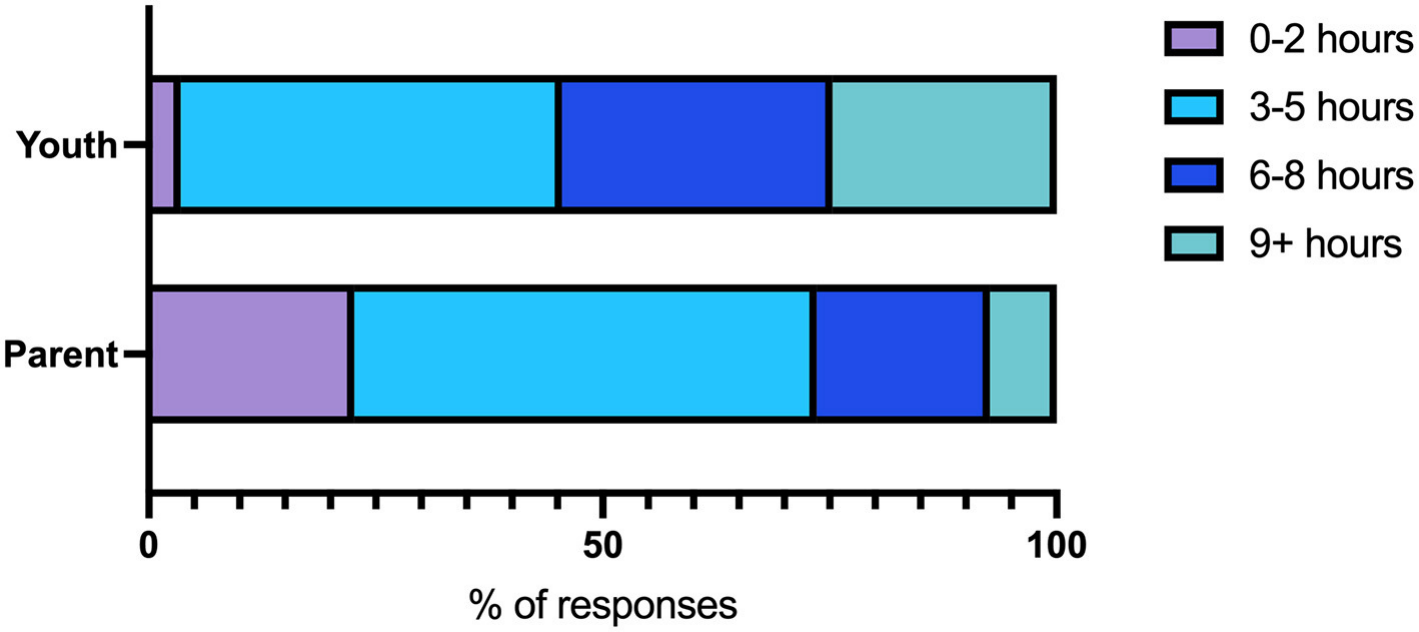
Number of hours of screen time within a 24-h period as reported by children and youth or parents.

Higher proportions of children and youth reported higher amounts of screen time (≥6 h) compared to parents (Figure 6). Most parents and youth reported that screen time was higher than before the pandemic started (Table 3).

### Open-Ended Questions About Return to School

Most parents reported that their child missed friends and social interactions (*n* = 549) when schools were closed to in-person learning. It was also reported by parents that students missed teachers (*n* = 122), in-person learning (*n* = 111), routine of attending school (*n* = 74), physical education, sport or physical activity (*n* = 56), or extra-curricular activities (*n* = 53). Students most frequently reported that they missed friends and social interactions most when school closed to in-person learning (*n* = 158) in addition to missing in-person classes or learning (*n* = 72), extra-curricular activities (*n* = 31), school routine (*n* = 25), and/or leaving home to attend school (*n* = 16).

Many parents commented that their child was looking forward to social interactions (*n* = 479) when returning to in-person learning at school. In addition, parents reported that children were looking forward to in-person learning (*n* = 119), routine of attending school (*n* = 86), interacting with teachers (*n* = 62), sport or physical activity (*n* = 40), and/or extracurricular activities (*n* = 16). Some parents (*n* = 52) reported that their child was not looking forward to returning to school. About half of students (*n* = 147) reported looking forward to social interactions. Students also reported looking forward to in-person learning (*n* = 43), routine of attending school (*n* = 29), sport or physical activity (*n* = 18), leaving home for school (*n* = 18), extra-curricular activities (*n* = 9) and their graduation year (*n* = 5). Some students (*n* = 34) reported that they were not looking forwarding to anything about going back to school.

Parents reported that they perceived their children were worried about the following in relation to returning to school: COVID-19 spread or outbreaks (*n* = 249), COVID-19 restrictions and rules (e.g., masks, physical distancing, class cohorts) (*n* = 213), social interactions (e.g., seeing friends in other classes or grades) (*n* = 99), academics (e.g., school work, workload or being behind) (*n* = 47), schools closing again (*n* = 39), new school or teacher (*n* = 33), no social or extra-curricular activities (*n* = 22), being away from home/ family (*n* = 22), and student mental health (*n* = 7). Some parents (*n* = 56) reported that their child was not worried about returning to school. Most worries reported by youth were specifically related to COVID-19. For example, 99 students were worried about catching or spreading COVID-19, while 77 students were worried about COVID-19-related practices. Youth also identified they worried about the following: schoolwork or workload (*n* = 44), social interactions (*n* = 27) and/or their mental health (*n* = 14). Twelve students were not worried about anything related to going back to school.

Many parents (*n* = 230) reported that their child hoped social interactions would be the same when they returned to in-person learning. In addition, parents reported that their child hoped outdoor or play time and recess (*n* = 104), extra-curricular activities (*n* = 90), and in-person classes and learning (*n* = 87) would be the same when they returned to in-person learning. Students hoped teaching and learning (*n* = 43), friends and social interactions (*n* = 42), extra-curricular activities (*n* = 40), and the school schedule or routine (*n* = 35) would be the same when they returned to in-person learning.

Some parents (*n* = 88) reported that their child hoped nothing would be different about school in the Fall. Parents also reported that their child hoped the following would be different: appropriate COVID-19 rules and regulations (e.g., mandatory masks, hand washing, physical distancing) (*n* = 65), academic support and/or support from teachers (*n* = 45), different school routine or schedule (e.g., virtual or blended learning) (*n* = 42), different social opportunities/ interactions (e.g., less bullying, and social interaction with other student “bubbles”) (*n* = 40). Youth reported that they hoped the following would be different when they returned to in-person learning: COVID-19 precautions (*n* = 65), more support for students (*n* = 22), options for online learning (*n* = 35), student behavior (*n* = 8), and/or school structure (*n* = 8).

### Access to Support and Resources

Twenty-one percent of parents and 29.8% of youth reported that they accessed supports/resources related to mental health, nutrition, or physical activity during the pandemic. In open-ended responses, parents described a variety of supports or resources accessed by their child: mental health support (*n* = 70), sport or physical activity (*n* = 59), and/or other healthcare providers (*n* = 18). Youth reported accessing the following supports: mental health supports (e.g., psychologist, Kids Help Phone), sport or physical activity (*n* = 18), and/or other healthcare providers (*n* = 5). Parents identified a variety of additional resources that would have been helpful for their child during the pandemic: academic and school support (e.g., online learning, guidance counselors) (*n* = 137), mental health support (*n* = 31), connection to peers (*n* = 31), sport or physical activity (*n* = 20), material resources (e.g., internet, school supplies) (*n* = 16), and/or childcare (*n* = 3). Twenty-one students commented that additional supports from their schools or teachers would have been helpful, in addition to formal mental health supports (*n* = 16), sport or physical activity (*n* = 8) and/or material resources (e.g., cleaning products or wipes) (*n* = 3).

## Discussion

This study investigated the health and well-being of children and youth during the first wave of the COVID-19 pandemic in Nova Scotia, as reported by parents and youth. Data were collected in August 2020, just before in-person learning resumed (September 2020). Previous themes that have emerged in studies of children/youth during the pandemic are feeling helpless to protect loved ones, challenges in maintaining friendships remotely, missing out on academic or extracurricular celebrations and milestones, and coping with school closures as they adapted to virtual learning (16). In our study, parents and youth responded that their children or they, respectively, felt bored during the COVID-19 pandemic, with most participants reported missing important life events, such as graduation ceremonies. Parents and youth both expressed that youth missed their friends and social interactions, in-person learning and extra-curricular activities while away from school. Parents and youth reported that youth were worried about COVID-19 spread or outbreaks, new COVID-19 rules, and the workload of returning to in-person learning.

Both parents and youth reported feeling bored and safe during the pandemic. In a previous study, 80% of Canadian youth reported feeling bored during the pandemic (17). Parents also reported their child felt lonely and this may reflect that these children were younger and not able to keep in touch with friends as easily as older children who maintained connections virtually. Canadian parents of 2–18-year-old children were surveyed and almost half reported that their children's mental health status (depression, anxiety or irritability/conduct problems) deteriorated during the first wave of the COVID-19 pandemic, and that deterioration in depression or anxiety was associated with greater stress for social isolation. In the same study, 35–45% of 10–12 year-old children and 38–48% of 13–18 year-old adolescents reported that their mental health status deteriorated during the pandemic (18). In our study, many youths reported feeling depressed and/or worried and many parents reported that youth felt anxious. The differences between parent and youth responses observed by Cost et al. (18) and in our study highlight the importance of surveying both parents and children once children are old enough to accurately self-report their thoughts and feelings.

In our study, parents and youth shared that social connections with parents, family, friends or pets contributed to them feeling good during the pandemic. The strategies identified by youth in our study were similar to previous research that identified some youth were spending more time with their families, having more fun together or having more meaningful conversations during the pandemic (10). A study from Ontario reported that youth found connecting with friends or family remotely, and/or pets were beneficial during lockdown (19). In UNICEF's COVID-19 survey, youth shared that more time with family, more personal time, the opportunity to slow down and take a break from being busy, more sleep and gratitude for important things in life were all positive outcomes of the COVID-19 pandemic (10). The youth in our study most frequently reported missing friends and social interactions at school and were most looking forward to social interactions when returning to in-person learning. Previous research has also reported that youth missed friends and social interactions during the pandemic, but technology allowed them to maintain some social connection (10, 17). Hawke et al. (19) also reported that youth and young adults could have benefited from more mental health, financial and social support during the pandemic. Similarly, both parents and youth in our study reported that access to mental health services, academic support, peer support or physical activity opportunities would have been helpful for children and youth during the pandemic. Youth and parents in our study reported that they were worried about COVID-19 outbreaks when returning to school, a worry previously shared by many youth and adults in another Canadian survey (17). It is important to recognize that in-person learning contributes to overall student health and well-being. In September 2020, the Canadian Pediatric Society issued an advocacy statement urging governments to keep schools open (with appropriate COVID-19 risk mitigation strategies) to support child development and mental health. More specifically, schools were encouraged to maintain and adapt non-academic programs (i.e., art, nutrition, physical activity), ensure in-school health supports (i.e., psychologists, occupational therapists) are available and to seek perspectives from students on their school experience (20). The perspectives offered by parents and youth in our survey reinforce these recommendations.

We also observed differences between parent and youth reports of healthy behaviors. For example, more parents vs. youth reported their children were sleeping more than before the pandemic. In our study, most parents also reported their child was engaging in more screen time, although youth reported higher hours per day of screen time than parents reported. Almost half of parents and youth reported that children and youth were doing less physical activity than before the pandemic, despite most reporting feeling safe outdoors and having similar access to outdoor spaces. In a national study of Canadian children and youth, parents reported significant declines in outdoor physical activity and sport, increases in recreational screen time and social media use, and increases in sleeping time during the COVID-19 pandemic (21).

Our study has several strengths. It is one of few studies that reports on the well-being of children and youth from the perspectives of both parents and youth themselves at a specific point in time during a global pandemic. The inclusion of youth voice allowed us to understand how the pandemic affected youth directly. Throughout the pandemic, the lives of children and youth were drastically changed, and will continue to be affected during the recovery phase. It has been suggested that youth be engaged in developing creative solutions to the pandemic and that they are essential in restructuring policies, systems, workflows and communities affected by COVID-19 (16). The youth in our study identified what they were worried about, and what they hoped would be similar or different on the return to in-person school. For example, some students were worried about the workload of returning to in-person learning full-time after time away and some students hoped there would be more support for students upon their return. These concerns could have impacted strategies about re-opening to best support students. A limitation of our study is that participants were not a random sample of Nova Scotia families; participants were recruited through social media and through mailing lists of youth serving organizations and not via a specific sampling strategy. For example, our sample predominantly lived in urban areas, in households with two parents, and annual family income was >$50,000, suggesting our results may not be applicable to all Nova Scotian families. Our survey was designed specifically for this study and its external validity has not been examined with other samples. Furthermore, public health restrictions varied regionally and our sample may not be generalizable to families in other jurisdictions. In addition, our parent and youth responses were not paired so comparisons between these groups reflects two unique samples with different demographics. There is also limited reliability of our self-reported physical activity levels as evidence suggests children and parents may over-estimate physical activity levels when compared to objective measures (22).

Children and youth in Nova Scotia, and globally, have demonstrated great resilience to the challenges posed by COVID-19. While parents and youth reported many negative feelings since the pandemic started, many also reported positive feelings; this has been a silver lining to the COVID cloud. It was evident that children and youth missed important life events because of the pandemic and missed their friends while schools were closed to in-person learning. A pandemic recovery plan must prioritize children's health and well-being, including innovative programs and policies to support physical and mental health. For example, both arts engagement and physical activity participation can be beneficial to mental health and should be promoted to children and youth as either in-person or virtual leisure-time activities depending on current public health restrictions (23, 24). Future research is needed to examine the health and well-being of children and youth now that they have returned to school with COVID-19 related restrictions and policies.

## Data Availability Statement

The raw data supporting the conclusions of this article will be made available by the authors, without undue reservation.

## Ethics Statement

The studies involving human participants were reviewed and approved by Dalhousie University Research Ethics Board. Written informed consent to participate in this study was provided by the participants' legal guardian/next of kin.

## Author Contributions

HC conducted the data analysis, populated data tables, created figures, interpreted the data, and drafted the initial draft of the manuscript. SK and CF conceptualized the study design, drafted the survey, oversaw the acquisition, analysis, and interpretation of data, and reviewed and approved the final version of the manuscript.

## Funding

HC received salary support from the Dalhousie University Healthy Populations Institute and the Dalhousie Medical Research Foundation to complete this work.

## Conflict of Interest

The authors declare that the research was conducted in the absence of any commercial or financial relationships that could be construed as a potential conflict of interest.

## Publisher's Note

All claims expressed in this article are solely those of the authors and do not necessarily represent those of their affiliated organizations, or those of the publisher, the editors and the reviewers. Any product that may be evaluated in this article, or claim that may be made by its manufacturer, is not guaranteed or endorsed by the publisher.
